# Food marketing

**DOI:** 10.1093/eurpub/ckac129.003

**Published:** 2022-10-25

**Authors:** A Garde

**Affiliations:** School of Law and Social Justice, University of Liverpool, Liverpool, UK

## Abstract

The Commission has established a joint action with Member States through the Best ReMaP joint action (2020-2023) to facilitate the effective implementation of the Audiovisual Media Services Directive which, in its revised version, calls on Member States to ensure that codes of conduct protect children from exposure to unhealthy food marketing. This provision itself highlights the tension between, on the one hand, the desire to limit the exposure of children to harmful food marketing and, on the other, the likelihood of achieving this objective through the implementation of codes of conduct.

**Speakers/Panellists:**

**Nikolai Pushkarev**

Healthy Environments & EPHA Policy, European Public Health Alliance, Brussels, Belgium

Contact: nikolai.pushkarev@epha.org

EPHA will present the perspective of the public health advocacy community, and discuss some of the initiatives civil society are engaged in to help the EU move from rhetoric to practice.

